# Using a population health management approach to enroll participants in a diabetes prevention trial: reach outcomes from the PREDICTS randomized clinical trial

**DOI:** 10.1093/tbm/ibab010

**Published:** 2021-03-02

**Authors:** Kathryn E Wilson, Tzeyu L Michaud, Fabio A Almeida, Robert J Schwab, Gwenndolyn C Porter, Kathryn H Aquilina, Fabiana A Brito, Caitlin A Golden, Emily V Dressler, Carol A Kittel, Lea N Harvin, Ashley E Boggs, Jeffrey A Katula, Paul A Estabrooks

**Affiliations:** 1 Department of Kinesiology and Health, College of Education and Human Development, Georgia State University, Sports Arena, Atlanta, GA, USA; 2 Center for the Study of Stress, Trauma, and Resilience, College of Education and Human Development, Georgia State University, Atlanta, GA, USA; 3 Department of Health Promotion, College of Public Health, University of Nebraska Medical Center, Omaha, NE, USA; 4 Center for Reducing Health Disparities, College of Public Health, University of Nebraska Medical Center, Omaha, NE, USA; 5 Internal Medicine Division of General Medicine, University of Nebraska Medical Center, Omaha, NE, USA; 6 Department of Biostatistics and Data Science, Wake Forest School of Medicine, Winston-Salem, NC, USA; 7 Department of Health and Exercise Science, Wake Forest University, Winston-Salem, NC, USA

**Keywords:** Representativeness, Diabetes prevention program, Implementation, Feasibility, EHR

## Abstract

Population health management (PHM) strategies to address diabetes prevention have the potential to engage large numbers of at-risk individuals in a short duration. We examined a PHM approach to recruit participants to a diabetes prevention clinical trial in a metropolitan health system. We examined reach and representativeness and assessed differences from active and passive respondents to recruitment outreach, and participants enrolled through two clinical screening protocols. The PHM approach included an electronic health record (EHR) query, physician review of identified patients, letter invitation, and telephone follow-up. Data describe the reach and representativeness of potential participants at multiple stages during the recruitment process. Subgroup analyses examined proportional reach, participant differences based on passive versus active recruitment response, and clinical screening method used to determine diabetes risk status. The PHM approach identified 10,177 potential participants to receive a physician letter invitation, 60% were contacted by telephone, 2,796 (46%) completed telephone screening, 1,961 were eligible from telephone screen, and 599 were enrolled in 15 months. Accrual was unaffected by shifting clinical screening protocols despite the increase in participant burden. Relative to census data, study participants were more likely to be obese, female, older, and Caucasian. Relative to the patient population, enrolled participants were less likely to be Black and were older. Active respondents were more likely to have a higher income than passive responders. PHM strategies have the potential to reach a large number of participants in a relatively short period, though concerted efforts are needed to increase participant diversity.

**Implications**

**Practice**: The use of population health management (PHM) approaches may be a practical way to inform clinical referral processes for diabetes prevention programs.

**Policy**: Policymakers who want to decrease the burden of clinical referral for diabetes prevention programs while maximizing reach, efficiency, and accuracy in patient identification and program implementation should explore sustainable PHM approaches.

**Research**: A PHM approach is supported for the accrual of large numbers of patients over a relatively short period of time with minimal burden on clinic staff.

## INTRODUCTION

The Diabetes Prevention Program (DPP) demonstrated that intensive behavioral counseling for lifestyle management reduced the incidence of Type 2 diabetes by 58% [1]. This success led to several adaptations of the DPP lifestyle intervention to translate these findings into sustainable practice [2]. Despite the effectiveness of these adaptations, the impact of these approaches is dependent on engaging individuals at risk for diabetes to participate—which has been challenging [3]. For example, a recent study of over 50,000 U.S. adults found that 73.5% of those with diagnosed prediabetes reported receiving advice and/or referrals for diabetes risk reduction from their healthcare professional. Of those, 35%–76% reported engaging in health behavior change or a diabetes prevention program in the past year. Importantly, only 33%–40% of those that reported receiving recommendations from healthcare professionals engaged in a diabetes prevention program, and overall participation in diabetes prevention programs has been exceedingly low (https://pubmed.ncbi.nlm.nih.gov/31074808/).

Population health management (PHM) principles may provide an opportunity to address the challenges of reaching people at risk for Type 2 diabetes. PHM includes a focus on the proactive identification of patients that could benefit from prevention or self-management interventions, technology-supported patient–provider communication, and linking patients to evidence-based approaches to improve health [4]. PHM approaches may be more efficient in engaging patients in prevention programs when compared to the traditional approach, for example, those that use office visits and physician referrals to accrue program participants [4]. Though provider referral plays a role in patient engagement [5], a consistent barrier to this approach has been the added time necessary for physicians to familiarize themselves with available programs and the burden of remembering to refer patients to the wide variety of programs available. The PHM approach is a viable workaround for these issues that may streamline the process of program delivery to those most in need.

The purpose of this study was to assess the reach of a clinical trial aimed at the prevention of Type 2 diabetes in adults with prediabetes, delivered using a PHM approach within a metropolitan healthcare system. Furthermore, we aimed to describe the proportion and representativeness of participants engaged at various points in the iterative recruitment process. In addition to the primary aims, we conducted exploratory analyses to address two secondary aims. Evidence suggests that participants that proactively respond to study recruitment material may be less representative of the overall population [6]. As such, we examined the possibility of differences in demographic, behavioral, and psychosocial factors and markers for disease risk among participants who actively responded to written recruitment materials prior to telephone outreach compared to participants recruited in response to telephone outreach. Finally, we conducted an opportunistic analysis to assess the impact of a shift in screening strategies from a pragmatic, less clinically sensitive approach that minimized participant burden to an approach that was more clinically rigorous but more burdensome for participants.

## METHODS

Recruitment data were collected from the PREDICTS trial: a hybrid effectiveness-implementation Type 1 single-blind randomized controlled trial (RCT) to determine the effectiveness of a digital DPP in reducing body weight and HbA1c. The trial protocol is described elsewhere [7] and was approved by the University of Nebraska Medical Center Institutional Review Board and Western Institutional Review Board and is registered at clinicaltrials.gov (NCT03312764).

### Recruitment and enrollment

Potential participants were identified through the electronic health record (EHR) with an automated search for ICD-9 and ICD-10 codes to capture patient records suggesting possible eligibility based on eligible age, body mass index (BMI), and, if available, a recent hemoglobin A1c laboratory test indicative of prediabetes. [7] The EHR analysis then removed records with ICD-9 and ICD-10 codes that matched study exclusion criteria (e.g., diabetes). [7] After the EHR identification, a total of eight primary care clinics affiliated with the Nebraska Medicine healthcare system were recruited to the study as recruitment and assessment sites; these eight clinics had the largest volume of potential participants identified in the EHR. Primary care physicians (PCPs) were asked to review a list of potentially eligible participants under their care to identify anyone who should not be included for reasons not captured in the EHR selection process. All persons approved for inclusion by their PCP received a personalized invitation letter from their PCP, including a postage-paid “opt-out” postcard and study contact number.

People could proactively engage in the screening process by using the contact information provided in the invitation. Those who contacted the staff to either opt out or be screened were classified as “active responders.” Those who did not actively respond were classified as “passive responders.” Passive responders received up to seven outreach telephone calls, from research staff, over 2–3 weeks to assess interest and screen for eligibility. Individuals who returned the “opt-out” postcard or contacted the “opt-out” telephone line after telephone outreach was initiated were still classified as “passive responders” as they required additional prompting to respond to the initial contact attempt. People who expressed interest in participating provided verbal informed consent for the phone screening immediately prior to being screened.

Staff conducting telephone screening were instructed to prioritize people identified as a racial/ethnic minority and those aged 65 years and older. We intended to sample a racially/ethnically representative sample of the healthcare system population, which was approximately 15% of the potentially eligible patient population. Furthermore, we aimed to recruit up to 20% of the sample to be aged 65 years or older to allow for subgroup analysis of intervention effectiveness. People potentially eligible from telephone screening were invited to an in-person screening to confirm eligibility based on an HbA1c test result between 5.7% and 6.4%. In-person screenings took place at the eight participating clinics. Participants were provided with the informed consent document at least 48 hr prior to their visit, and a trained research staff member provided a consent presentation and comprehension check prior to participant consent and obtained consent at the beginning of each in-person screening visit.

Initially, a point-of-care (POC) test (A1CNow+, Professional Multi-test HbA1c system; Polymer Technology System, Inc., Indianapolis, IN) was used to assess HbA1c. Those with POC HbA1c in the prediabetes range were considered eligible for participation, enrolled in the study, and underwent baseline assessment [7], which included a blood draw for a laboratory-derived HbA1c (i.e., the primary study outcome). However, after 6 months of study recruitment, a high proportion of false-positive POC results (52%) were observed compared to the baseline laboratory tests. The screening protocol was, therefore, adjusted to use a laboratory-confirmed HbA1c of 5.7%–6.4% to determine prediabetes eligibility. With 116 participants enrolled with a baseline HbA1c outside of the prediabetes range, the trial sample size was increased from 482 to 599 to retain sufficient power to analyze HbA1c changes among those with confirmed prediabetes. Participants with false-positive POC HbA1c were allowed to continue with the trial but were excluded from final analyses. This protocol change resulted in an additional study visit to complete baseline data collection after prediabetes was confirmed with the lab test.

The two study conditions (a) small group, in-person class and (b) a digital DPP are described in more detail elsewhere [7]. All investigators and staff taking measurements at the assessment visits were blinded to intervention assignment. The project managers, statisticians, and research assistants were not involved in measurement, and the study physician was unblinded. Recruitment phone calls began on December 4, 2017, and ended on February 18, 2019. The final participant was enrolled on March 16, 2019.

### Measures

Reach was operationalized as the proportion of patients who enrolled in the study relative to (a) the sample of participants who were screened for eligibility and (b) the total participant pool across adopting clinics [8]. Representativeness was assessed in the context of the demographic landscape of the region as well as that of the patient population and was defined as the comparability between the study sample and the surrounding and intended population as described by census data [9] and results from the Nebraska Medicine EHR system query, respectively. Whereas the pool of individuals who were EHR eligible (EHR pool) was restricted to patients aged 19 and older, at risk of diabetes, with a BMI of 25 kg/m^2^ or greater, and had no illnesses that could potentially prevent them from participating in the trial, the census data cannot be filtered for those qualifiers, and, thus, the denominator in the obesity rate calculation in the EHR eligible population is limited to overweight or obese individuals, whereas the denominator of obesity rate in the census data includes all weight categories.

Participants self-reported demographic characteristics of age, gender, race, ethnicity, marital status, education level, employment, yearly household income, type of healthcare insurance, and household size. Measured diabetes risk factors included HbA1c, body weight, BMI, blood lipids, and blood pressure. Psychosocial measurements included satisfaction with body function and appearance [10], subjective well-being (WHO-5) 11, quality of well-being [12], mental health outcomes (Patient Health Questionnaire screener; PHQ-4) 13, perceived stress [14], loneliness [15], self-efficacy for weight-loss-related behaviors [16], social provisions for behaviors related to weight loss [17], medication adherence [18], health literacy [19], absenteeism/presenteeism at work (Stanford Presenteeism Scale [20]; WHO Health and Work Performance questionnaire [21]), and self-reported healthcare utilization [22,23]. Participants were classified as either meeting physical activity recommendations or not based on responses to the Godin–Shephard Leisure Time Physical Activity Questionnaire [24]. The “Starting the Conversation” Brief Dietary Assessment was used to assess nutritional intake [25]. The Medical Outcomes Survey Sleep Scale [26] was used to capture hours of sleep per night and the Berlin Questionnaire was used to assess the risk for sleep apnea [27].

### Statistical analysis

In order to determine the representativeness of PREDICTS participants, demographic characteristics of those enrolled in the study were compared to (a) city-level census estimates [9] and (b) the EHR population. One-sample *t*-test or one-sample test of proportion examined group differences for demographic characteristics of the sample compared to census data and the EHR pool for continuous and categorical outcomes, respectively. A one-sample Wilcoxon signed-rank test was used to compare the median age of the participants to that of available census data. Proportional reach and demographic representativeness were examined for each iterative stage of the recruitment process (i.e., proportion and representation of patients identified in the EHR audit screened by telephone, screened in-person, and enrolled). Reach and representativeness of the sample were also assessed relative to the EHR population representative of each clinic.

Two-sample tests of proportion were used to determine whether participation rates differed according to (a) response type (active vs. passive responders) and (b) HbA1c screening protocol (POC vs. venipuncture). Independent samples *t*-test and chi-squared tests were used within the final enrolled sample to test whether subgroups (i.e., active vs. passive responders; POC vs. venipuncture screening protocols) differed demographically or with respect to markers of disease risk. Finally, independent samples *t*-tests and chi-squared analyses were used to examine behavioral and psychosocial characteristics between active and passive responder subgroups. In the case of significant heteroscedasticity, as indicated by Levene’s test of equality of variances, adjusted *t*-tests are reported.

## RESULTS

### Study reach

**Fig 1:**
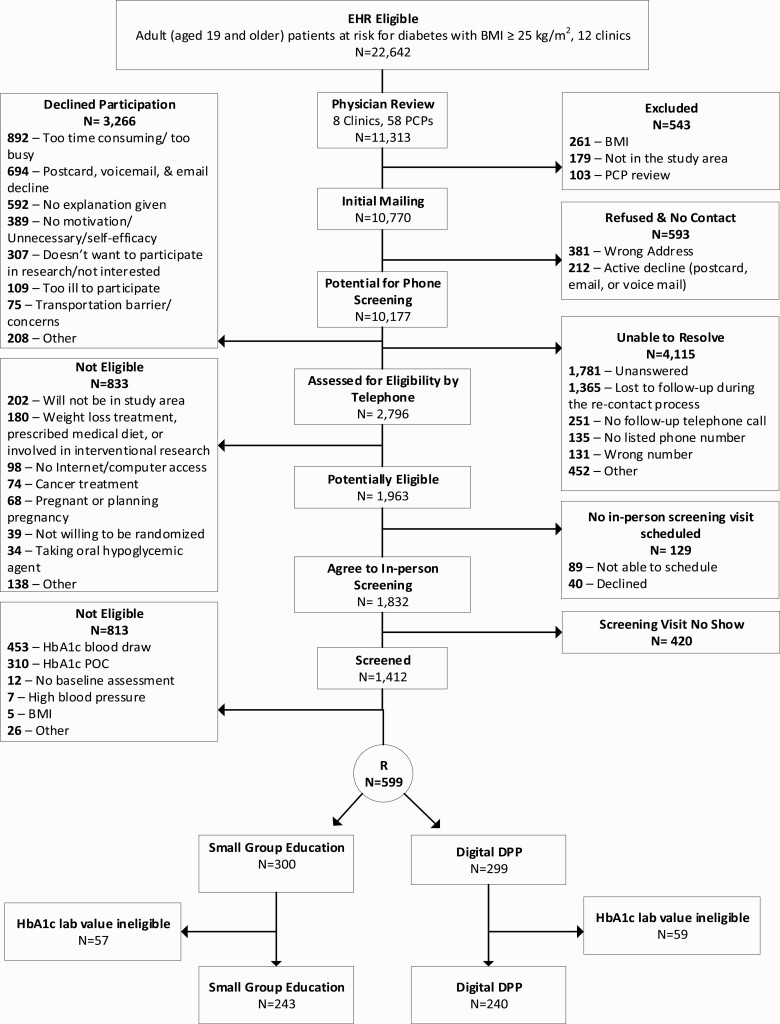
Recruitment and enrollment flow diagram.

The recruitment flow is presented in Fig. 1. A total of 22,642 potential participants were identified in the EHR across two time points during the 1 year recruitment period. Of these, 11,313 received care in the eight participating clinics and were reviewed by their PCPs for medical clearance. A total of 103 patients were excluded by their PCPs, 261 patients were excluded for a BMI below 25 kg/m^2^, and 179 patients had moved away from the study area. Recruitment mailers were sent to 10,770 patients, of which 381 (4%) were returned due to having the incorrect address, and 212 (2%) actively declined using the opt-out postcards. Among the remaining 10,177 potential participants, 2,796 (28%) completed the telephone screening, 3,266 (32%) declined to complete the screening, and 4,115 (40%) were not screened but did not definitively decline participation. The most frequently reported reasons for declining telephone screening were being too busy (27%) and no motivation to participate (12%). Of those not screened/did not decline, the majority were due to initial (18%) or follow-up (13%) calls being unanswered. Of those that completed telephone screening, 1,961 were identified as potentially eligible and 1,412 attended a screening session. Forty-five percent of those screened were eligible (*n* = 630) and 599 people were enrolled—reflecting 5.8% of the patient population who received an invitation mailer (599/10,770) and 21% of those who underwent telephone screening (599/2,796). A total of 5,218 hr were spent on the recruitment process as a whole, excluding the processes conducted by physicians (e.g., review of patient list). Recruitment mailer and phone calls alone required about 57 hr per week.

### Sample representativeness

Table 1 presents census demographic data of Omaha, the sampling pool generated from Nebraska Medicine EHR records, and the study sample at various stages in the recruitment process. The study yielded a significantly greater proportion of individuals who were obese, female, 65 or older, and White relative to the census and EHR population. African Americans were underrepresented compared to census and EHR data and Latinx were underrepresented compared to census data.

**Table 1:** Overall reach and representativeness of PREDICTS participants according to stage of recruitment

	Census data^a,b,c^ (1)	EHR eligible (2)	Recruitment mailers sent	Telephone screened	In-person screened	Eligible	Enrolled (3)	Group Comparison^d^ (1) vs. (3)	Group Comparison^e^ (2) vs. (3)
*N*	468,286	22,642	10,770	2,796	1,412	630	599		
% obese	33	60	85	87	87	91	92	31.03*	16.36*
% female	51	56	60	65	63	61	61	5.30*	2.68*
*M* (*SD*), age	35.1	49.0 (15.6)	49.6 (15.1)	51.7 (14.6)	53.2 (13.4)	53.2 (13.4)	55.8 (12.6)	20.93 (367)*	11.48 (598)*
% ≥65 years	12	19	19	22	22	25	25	9.49*	4.12*
% Caucasian	78	83	80	83	90	89	90	7.18*	4.82*
% African American	12	11	14	12	7	8	7	−4.31*	−3.38*
% Hispanic/Latino	14	4	5	5	3	3	3	−7.33*	−1.13

*SD* standard deviation.

^a^ https://www.census.gov/quickfacts/omahacitynebraska.

^b^ https://factfinder.census.gov/faces/nav/jsf/pages/community_facts.xhtml?src=bkmk.

^c^ https://www.americashealthrankings.org/explore/annual/measure/Obesity/state/NE.

^d^Values for group comparisons are one-sample *z*-tests of proportion for categorical variables and Wilcoxon signed-rank test for age.

^e^Values for group comparisons are one-sample *z*-tests of proportion for categorical variables and one-sample *t*-test for continuous variables. Values for age in the Census data column are reported medians.

*p ≤ .01.

### Reach by clinic

Table 2 shows study reach for the eight adopting clinics (A–H). Total physicians at each clinic ranged from 3 to 36 with a median of five physicians per clinic. Between 88% and 100% of the respective EHR pool for each clinic were sent a recruitment mailer. Across clinics, between 21% and 32% of patients participated in the telephone screening, and an average of 53% was potentially eligible and attended an in-person screening. After the in-person screening, an average of 48% was eligible across clinics. Overall, between 14% and 35% of individuals screened by telephone from each clinic were enrolled.

**Table 2:** Study reach and sample representativeness by adopting clinics

Clinic	Number of physicians engaged^a^	EHR eligible *N* (%)^b^	Recruitment mailers sent *n* (%)	Telephone screened *n* (%)	In-person screened *n* (%)	Eligible patients *n* (%)	Minority *n* (%)^c^	Older adults *n* (%)^c^	Female *n* (%)^c^	Reach by clinic *n* (%)^d^
A	5	1,340 (12)	1,175 (88)	380 (32)	190 (50)	90 (47)	9 (10)	18 (20)	44 (49)	89 (23)
B	9	1,514 (13)	1,478 (98)	401 (27)	196 (49)	102 (52)	1 (1)	21 (201)	68 (67)	102 (25)
C	6	631 (6)	631 (100)	153 (24)	53 (35)	27 (51)	8 (32)	6 (24)	19 (76)	25 (16)
D	3	1,006 (9)	990 (98)	208 (21)	128 (62)	71 (56)	5 (8)	14 (21)	39 (59)	66 (32)
E	4	1,020 (9)	1,000 (98)	248 (25)	143 (58)	91 (64)	1 (1)	22 (25)	57 (66)	87 (35)
F	5	777 (7)	759 (98)	194 (26)	120 (62)	47 (39)	0	15 (33)	30 (67)	45 (23)
G	36	4,532 (40)	4,249 (94)	1,075 (25)	497 (46)	169 (34)	23 (15)	47 (31)	79 (52)	153 (14)
H	3	493 (4)	487 (99)	137 (28)	85 (62)	33 (39)	1 (3)	7 (22)	32 (100)	32 (23)

Values in parentheses are proportions for which the denominator equals the *n* indicated in the preceding cell of the respective row, unless otherwise specified.

*EHR* electronic health record.

^a^Physicians engaged (*n* = 58) were not mutually exclusive across clinics.

^b^% of patients from each clinic relative to the total sampling pool identified in the EHR query.

^c^% of participants of the indicated classification relative to the total enrolled sample from the respective clinic.

^d^% of patients that completed phone screening who were subsequently enrolled.

### Active versus passive responder subgroup analyses

The majority (93%) of patients that received a recruitment mailer were classified as passive responders (*n* = 9,984). Of these, 26% (*n* = 2,603) agreed to a telephone screen when reached by telephone follow-up. After telephone screen, 69% (*n* = 1,795) were eligible for in-person screening; 71% (*n* = 1,269) attended in-person screening, 45% (*n* = 569) were found eligible, and finally, 95% of those who were found eligible (*n* = 542) were enrolled in the study. Among active responders (*n* = 405), 48% (*n* = 193) requested to participate in the telephone screening (whereas 52% actively responded to opt out of the study). Of those, 87% (*n* = 168) were potentially eligible, 85% of which (*n* = 143) participated in the in-person screening. A total of 61 (43%) screened eligible and 57 of them (93%) were enrolled in the study, representing 10% of the total enrolled sample. The proportion of total active (*n* = 405) and passive (*n* = 9,984) responders that were enrolled in the study (14% of active responders, and 5% of passive responders) was significantly different (*z* = 7.38; *p* < .01). The proportion of active (30%; *n* = 1,795) and passive (21%; *n* = 168) responders found potentially eligible via telephone screening that were enrolled in the study was also significantly different (*z* = 2.84; *p* < .01).

Demographic and physiological characteristics of the enrolled sample by active versus passive response are displayed in Table 3. The active responders had a higher baseline HbA1c (*t*(*df*) = 2.32(597); *p* = .02) and fewer persons living in the household than passive responders (*t*_adj_(*df*) = −3.23(85.5); *p* < .01). A significantly higher proportion of active responders reported an annual household income ≥$100,000 and a significantly lower proportion of active responders reported an annual household income <$50,000 compared to passive responders (χ ^2^(*df*) = 7.01(2); *p* = .03). A higher proportion of active responders reported having private health insurance (80.7%) compared to passive responders (65.1%; χ ^2^(*df*) = 6.36(2); *p* = .04). Otherwise, active and passive responder groups did not differ based on demographic characteristics, disease risk, or behavioral and psychosocial variables (Table 4).

**Table 3:** Baseline demographic and physiological descriptions of the sample according to recruitment and screening subgroups

	Total enrolled	Active response enrolled	Passive response enrolled	POC HbA1c screen enrolled	Lab HbA1c screen enrolled
*N* (%)	599	57 (10)	542 (91)	254 (42)	345 (58)
*n* (%)					
Female	368(61)	40(70)	327 (60)	140 (55)	228 (66)**
Caucasian	541(90)	51(90)	491 (91)	233 (92)	308 (89)
African American	39(7)	5(9)	34 (6)	13 (5.1)	26 (8)
Hispanic or Latino	20(3)	56(98)	521 (96)	8 (3)	11 (3)
Married	435(73)	42(74)	393 (73)	181 (71)	254 (74)
Employment status					
Employed	327(55)	32(56)	295 (54)	143 (56)	184 (53)
Retired	144(24)	16(28)	128 (24)	53 (21)	91 (26)
Other	128(21)	9(16)	119 (22)	58 (23)	70 (20)
Education					
High school or less	79 (13)	3 (5)	76 (14)	36 (14)	43 (13)
College (any)	369 (612)	35 (61)	334 (62)	153 (60)	216 (63)
Advanced degree	149 (25)	19 (33)	130 (24)	63 (25)	86 (25)
Annual income					
<$50,000	165 (278)	9 (16)	156 (29)*	72 (28)	93 (27)
$50,000 to $100,000	214 (36)	19 (33)	195 (36)	86 (34)	128 (37)
>$100,000	208 (35)	28 (49)	180 (33)*	89 (35)	119 (35)
Insurance					
Medicare/Medicaid	159 (27)	9 (16)	150 (28)	64 (25)	95 (28)
Private	399 (67)	46 (81)	353 (65)*	166 (65)	233 (68)
Other	34 (6)	1 (2)	33 (6)	19 (8)	15 (4)
*M* (*SD*)					
Age, years	55.6 (12.8)	57.6 (10.3)	55.2 (13.0)	53.3 (13.7)	57.0 (11.8)**
Persons per household	2.7 (1.5)	2.2 (1.0)	2.7 (1.5)*	2.8 (1.4)	2.6 (1.5)
Lab HbA1c	5.8 (0.3)	5.9 (0.2)	5.8 (0.3)*	5.7 (0.3)	5.9 (0.2)**
BMI, kg/m^2^	36.0 (6.4)	35.7 (6.7)	36.0 (6.3)	36.4 (6.3)	35.6 (6.4)
HDL mg/dL	49.2 (12.5)	50.2 (11.5)	49.1 (12.6)	48.2 (11.6)	50.0 (13.1)
LDL mg/dL	102.8 (32.1)	106.9 (29.7)	102.4 (32.3)	104.8 (32.9)	101.5 (31.5)
Triglycerides mg/dL	195.7 (15.9)	187.1 (95.3)	196.6 (117.9)	202.8 (128.8)	190.5 (105.2)
Systolic BP, mmHg	128.6 (15.1)	131.4 (14.6)	128.3 (15.1)	131.3 (16.8)	126.6 (13.4)**
Diastolic BP, mmHg	79.5(10.6)	75.9 (10.1)	79.8 (10.6)	83.1 (10.4)	76.8 (10.0)**

*SD* standard deviation.

*p < .05; **p < .01.

**Table 4:** Behavioral and psychosocial descriptions of the sample by recruitment and screening subgroups

	Total enrolled	Active response enrolled	Passive response enrolled
*N*	599	57	542
Health behaviors			
*N* (%) Inactive	393 (66)	41 (72)	352 (65)
*N* (%) current smokers	54 (9)	2 (4)	52 (10)
Sleep hours/night	6.7 (1.2)	6.6 (1.1)	6.7 (1.2)
Dietary intake	8.1 (2.5)	8.5 (2.5)	8.0 (2.5)
*N* (%) nonadherent to medication	217 (36)	27 (53)	190 (43)
Quality of well-being score	0.7 (0.1)	0.6 (0.1)	0.7 (0.1)
WHO-5 score	63.0 (18.5)	62.0 (18.6)	63.1 (18.5)
PHQ-4 score	2.1 (2.6)	2.4 (3.0)	2.1 (2.5)
Perceived stress	3.9 (2.8)	4.0 (3.1)	3.9 (2.8)
Loneliness	4.0 (1.5)	4.3 (1.6)	4.0 (1.5)
*N* (%) at risk for sleep apnea	371 (62)	39 (68)	332 (61)
*N* (%) low health literacy	55 (9)	2 (4)	53 (10)
Body satisfaction			
Body function	−0.8 (1.5)	−1.0 (1.6)	−0.8 (1.5)
Body appearance	−1.9 (1.6)	−2.1 (1.3)	−1.9 (1.3)
Social provisions			
Guidance	12.7 (2.5)	12.4 (2.7)	12.7 (2.5)
Worth reassurance	10.1 (2.1)	9.8 (2.4)	10.1 (2.1)
Social integration	11.6 (2.1)	11.2 (2.1)	11.7 (2.0)
Attachment	12.0 (2.6)	11.9 (2.9)	12.0 (2.6)
Nurturance	9.4 (2.4)	9.5 (2.5)	9.4 (2.4)
Reliable alliance	12.6 (2.4)	12.5 (2.6)	12.6 (2.4)
Weight-loss-related behavior self-efficacy			
Physical activity self-efficacy	78.7 (17.4)	76.0 (22.0)	78.9 (16.9)
Healthful eating self-efficacy	74.9 (18.4)	74.5 (20.2)	74.9 (18.2)
Weight-loss self-efficacy	77.9 (18.3)	76.9 (17.7)	78.0 (18.3)
Work absenteeism/presenteeism			
Hours worked past 28 days	154.1 (53.8)	160.3 (43.2)	153.5 (54.7)
Hours work missed past 28 days	−1.9 (47.7)	−1.9 (59.3)	−2.0 (46.4)
Stanford presenteeism score	25.8 (4.4)	26.1 (4.6)	25.8 (4.4)
Health care utilization score	7.3 (8.6)	6.3 (6.0)	7.4 (8.8)

All measures taken at baseline. Values are *M* (standard deviation) unless otherwise specified.

### POC versus venipuncture screening subgroup analyses

**Fig 2:**
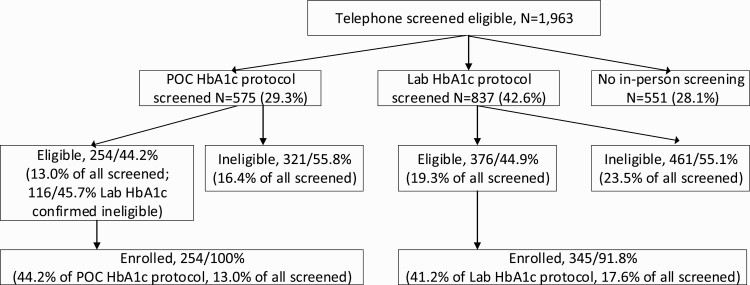
Flow of potential study participants through diabetes screening protocols from participant identification to study enrollment.

Figure 2 shows the enrollment flow for participants screened with POC versus venipuncture blood draw. In total, 575 (29%) were screened using POC, and 837 (43%) were screened by venipuncture test. The residual 551 (28%) did not attend an in-person screening. Of those screened using the POC HbA1c fingerstick, 254 (44%) were eligible and enrolled in the study. Of those screened by venipuncture blood draw, 376 (45%) were eligible and invited back for enrollment and baseline measures. The proportion of eligible participants identified by the two HbA1c testing methods were similar (*z* = −0.26, *p* = .80). Additionally, the proportion of patients enrolled under each screening protocol did not significantly differ (*z* = 1.12, *p* = .26). However, relative to the total sample of patients deemed potentially eligible, a smaller proportion of patients were found eligible by POC fingerstick than via venipuncture (65% vs. 77%, respectively; *z* = 5.78, *p* < .01).

**Fig 3:**
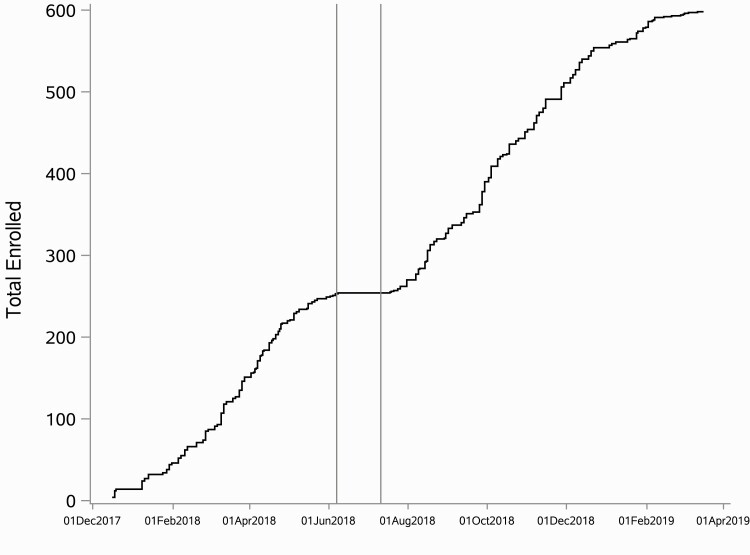
Participant accrual over time. Vertical lines delineate the duration of a pause (June 7 to July 10, 2018) in recruitment to accommodate the shift in screening procedures from point-of-care testing to venipuncture.

The subsample enrolled via venipuncture screening had a significantly higher proportion of females (χ ^2^(*df*) = 6.90(1); *p* < .01), higher average age (*t*_adj_(*df*) = −3.46(499); *p* < .01), higher baseline HbA1c (*t*_adj_(*df*) = 8.15(373.7); *p* < .01), and lower baseline systolic (*t*(*df*) = −7.45(596); *p* < .01) and diastolic (*t*_adj_(*df*) = −3.67(467); *p* < 0.01) blood pressure than those enrolled via POC fingerstick screening (Table 3). Figure 3 displays accrual over time with an indication of shift in protocol to venipuncture screening.

## DISCUSSION

Overall, the results of the PHM approach were promising with regard to reaching the intended at-risk population. The rate of participant accrual (40–50 per month) and the proportion of those screened eligible, who were eventually enrolled (95%), were superior to comparable clinical trials [28,29], though the representativeness of the sample compared to the surrounding area and to the total potentially at-risk patient population highlighted some areas for improvement.

The telephone screening eligibility rate of 45% compares favorably to the screening outcomes of the Diabetes Prevention Program Trial [26], which also used physician referral and telephone screening, but found only 20% to be potentially eligible. Our yield is also comparable to a recent effectiveness trial that used EHR data to identify potential DPP participants [30]. Our trial accrual rate of 13% enrolled relative to total attempted reach is comparable to their 11% using EHR across 12 clinics to identify potentially eligible patients.

Compared to both census and EHR data, our sample had a greater proportion of individuals who were obese, female, aged 65 years or older, and White. However, the oversampling of people 65 years or older was by design, as age is a known risk factor for prediabetes [31]. The trial also aimed to recruit a sample that was representative of the clinical Black and Hispanic/Latinx population and accrued 10% across all race/ethnic minority groups combined. Unfortunately, the proportion of African Americans and Hispanic/Latinx in the trial sample was lower compared to the regional census and, in the case of African Americans, lower than that of the EHR pool. Beyond including the largest primary care clinic with the highest proportion of racial and ethnic minorities (Clinic G), the trial did not include any specific strategies to engage individuals from these population groups. Therefore, specifying additional strategies to improve the engagement of minority participants is of critical importance for future work.

The vast majority of participants were passive responders (93%). Evidence suggests that study recruitment procedures that rely on participants to proactively engage contribute to poor sample representativeness, especially in socioeconomic diversity or disease risk [6, 29, 32–34]. Indeed, active responders in the trial were more likely to be in the highest income category and less likely to be in the lowest. Active responders were also significantly more likely to have private health insurance and smaller household size and had a significantly higher HbA1c compared to passive responders. A health equity lens is needed to ensure the successful engagement of participants from race/ethnic minority groups and lower socioeconomic status (SES) categories [35]. However, despite the socioeconomic differences, there were no behavioral or psychosocial differences between active and passive responders. While active follow-up approaches may be perceived as more time intensive and costly, it could take longer and cost more to use entirely passive approaches that require a greater volume of total outreach.

Finally, this study explored the effect of a mid-trial shift in screening protocols on the rate of participant accrual. Participant accrual did not suffer as a result of shifting protocols to a more invasive (i.e., venipuncture blood draw vs. fingerstick) and burdensome (i.e., two clinic visits instead of one) protocol. Under both protocols, accrual averaged at a rate of about 10 new participants per week. The elimination of false-positive screening with minimal clinical/demographic differences and without diminishing participant accrual speaks to the efficacy and feasibility of the more rigorous clinical screening method. Furthermore, the shift to venipuncture blood draw, with the exception of age (older), baseline HbA1c (higher, and expected), and blood pressure (lower), did not change the representativeness of the sample relative to demographic or other health indicators.

There are some limitations to consider when interpreting results. Though this evidence was generated in the context of a randomized controlled trial, the results are observational in nature. There was no manipulation or experimentation implemented to test the comparative effectiveness of the PHM approach relative to other methods. Furthermore, it remains unclear if the clinical system would be able to conduct the same intensity of follow-up implemented by the research staff in this study. With an aggressive approach to participant recruitment, we were able to accrue high numbers in a relatively short time period. It is noteworthy that, in application, many of the recruitment processes implemented in this trial are unnecessary for implementation in the clinic system. For example, in-person screening and associated activities would not be required if adopted in the clinic system. Comparisons between active and passive responders and between POC and venipuncture screening approaches were opportunistic. Thus, there is a possibility that subgroups differ in meaningful ways not captured here. This approach was limited in its ability to attract a representative population of race/ethnic minority participants, which highlights the need for focused efforts to ensure adequate representation. Finally, while results are generalizable to systems that use a comparable EHR system, overall generalizability is limited due to lower enrollment among minority participants.

Our findings support the use of PHM approaches to accrue a large number of patients over a relatively short period of time and, when considered in the context of other research, may be a practical way to inform clinical referral processes for diabetes prevention programs. Findings support the efficacy of PHM approaches to reach participants with efficiency and accuracy and reduce the potential burden on healthcare providers to supply referrals. Future studies should focus efforts to tailor/target their approaches to engage a higher proportion of minority participants. The approach we examined demonstrated strong reach, precision in patient identification, and feasibility for implementation with minimal burden on clinic staff. The continued use of PHM approaches leveraging EHR identification and clinician endorsement (but not referral) is supported. More work is needed to assess the feasibility of this approach for other organizations (i.e., the clinical system) without additional research personnel.
